# Local Cytotoxic Effects in Cobra Envenoming: A Pilot Study

**DOI:** 10.3390/toxins14020122

**Published:** 2022-02-07

**Authors:** Jing-Hua Lin, Wang-Chou Sung, Han-Wei Mu, Dong-Zong Hung

**Affiliations:** 1 Division of Toxicology, China Medical University Hospital, Taichung 40447, Taiwan; jh.cooltm@gmail.com (J.-H.L.); gackt0366@hotmail.com (H.-W.M.); 2 National Institute of Infectious Diseases and Vaccinology, National Health Research Institutes, Miaoli 35053, Taiwan; sung23@nhri.edu.tw

**Keywords:** cobra snakebite, dermonecrosis, cytotoxin A3, rapid diagnosis kit-ICT-Cobra

## Abstract

The cobra (genus *Naja* (*N.*)) is one of the most common venomous snakes. Due to its frequency and deadly complications of muscle paralysis, local necrosis, and chronic musculoskeletal disability, it should not be ignored. The pathology of devastating tissue destruction, even though specific antivenoms exist, is not fully clear. Here, we attempted to dig in envenomed tissues to study the clinical toxicology of cobra venom. Four cases of *N. atra* snake envenomation, in which the subjects developed advanced tissue injury, were involved in this study. We used enzyme-ligand sandwich immunoassay (ELISA) to assay the whole venom, cytotoxin A3 and short-chain neurotoxin (sNTX) in blood, bullae, wound discharge, and debrided tissue. We found that persistently high concentrations of venom and toxins, especially cytotoxin A3, were detected in bullae, wound discharge fluid and necrotic tissue of these patients even after large doses of specific antivenom treatment, and wide excision and advanced debridement could largely remove these toxins, lessen the size of necrosis, and promote wound healing. We also found that the point-of-care apparatus, ICT-Cobra kit, might be used to promptly monitor the wound condition and as one of the indicators of surgical intervention in cases of cobra envenomation in Taiwan.

## 1. Introduction

Venomous snakebites are a neglected tropical disease, and are estimated to involve millions of people, resulting in tens of thousands of deaths annually [1]. In addition to death, innumerable morbidities, such as loss of limbs due to excessive tissue/muscle damage, result in lifestyle disruption in these victims. Significant local tissue injuries are complicated by a variety of acute pathological alterations occurring at the site of venom injection that can advance to be destructive. Furthermore, tissue loss sequela is one of the detrimental outcomes of several types of snakebite envenomation, including species of the family Viperidae in Central and South America, such as genus *Bothrops* (*B.*), or genus *Naja* (*N.*) in the family Elapidae, also known as the cobra in Africa and Asia. Such alterations occur rapidly after the bite, and, depending on the amount of venom injected and the delay in antivenom treatment, they may result in permanent tissue damage and physical disability.

The mechanisms of local tissue damage caused by *B. asper* envenomation have been elucidated more clearly in vitro and in vivo, and toxins such as metalloproteinase (SVMPs) and phospholipase A_2_ (PLA_2_s) likely play major roles due to their toxic effects on the skin, subcutaneous tissues, vessels and muscle tissues and their sequential inflammatory reactions [2,3,4]. Bites on fingers with pressure bandaging or tourniquet application, in addition to relatively large doses of envenomation, were also found to be significant contributing factors to the local necrosis of viper snakebites [5,6]. Cobra snake bite-related tissue necrosis might share some but not all of the same mechanisms. A few cellular and animal studies have found that cytotoxins, major toxic components with abundances, ranging from 20% to as high as 70% of the total venom proteins [7,8,9,10,11], might play a major role in contributing to extensive tissue necrosis if envenomed by a cobra [12,13,14], but there is a lack of direct evidence in clinically envenomed cases.

*N. atra* is an important venomous snake in Asian regions, including Taiwan, and its envenomation may present with significant local tissue swelling and advanced necrosis [15,16,17,18,19]. Appropriate antivenom administration relies on early and correct identification of venomous snake species, and is likely to bring a good prognosis, allowing application of a rapid test, the ICT-Cobra kit, for the early diagnosis of cobra snakebites [15,19]. However, local tissue destruction accompanied by advanced necrosis remains a critical problem for cobra snakebites even after 15 vials of specific antivenom [15,18]. How to gain a greater insight into the presence of toxins in local necrotic lesion and their evolution after treatment, such as antivenom therapy and surgical intervention, have become an unavoidable challenge in clinic settings. Therefore, we attempted to collect wound discharge and debrided tissues from patients who were envenomed by *N. atra* for toxin analysis to expound the possible pathophysiology of local tissue destruction and investigate the feasibility of ICT-Cobra kit in monitoring the wound condition. The results would be helpful in drawing up the rational countermeasures in handling and/or preventing sequelae of tissue destruction in cases of cobra snakebites.

## 2. Results

**Figure 1:**
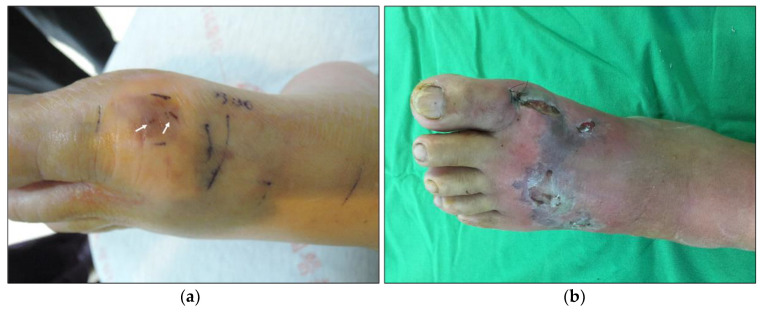
The typical wound progression of severe cobra envenomation on the foot of a 68-year-old female. (**a**) Nine hours after the snake bite. Fang marks (white arrow) were found on the lateral aspect of the first metatarsal joint of the left foot, and the injuries extended forward to the toes and backward to the instep with swelling and erythematous changes. The demarcated bruising skin indicated the possible range of subcutaneous necrotic change. No bullae formation was noted at this time; (**b**) Five days later. Increased erythematous swelling extended over the ankle joint, and worsened dermonecrosis was noted across the instep.

**Figure 2:**
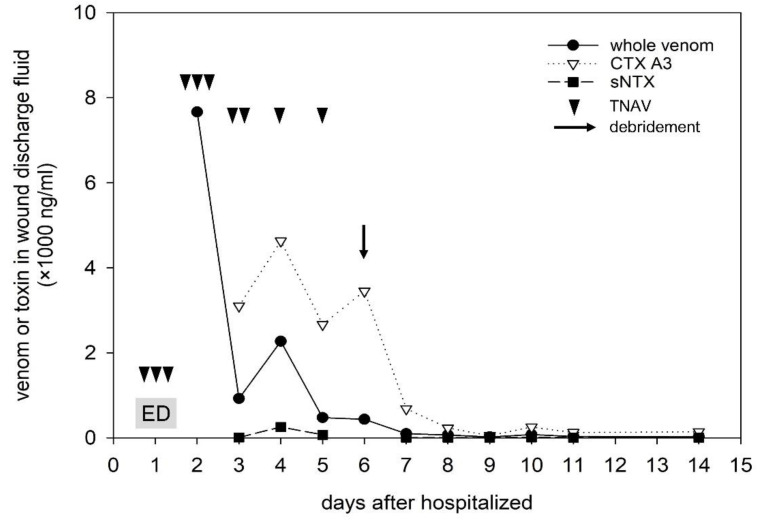
The evolution of the whole venom, CTX A3, and sNTX concentrations in the local wound discharge collected from the 68-year-old female. The data on Day 2 is the venom concentration of the vesicle fluid of bulla that developed about one day after being bitten. Venom concentration decreased significantly after the specific antivenom was administered. A relatively high concentration of CTX A3 was detected from the local wound discharge and persisted until advanced debridement on Day 6. ED: emergency department; TNAV: bivalent antivenom for *B. multicinctus* and *Naja atra*, produced by Taiwan Centers for Disease Control.

**Figure 3:**
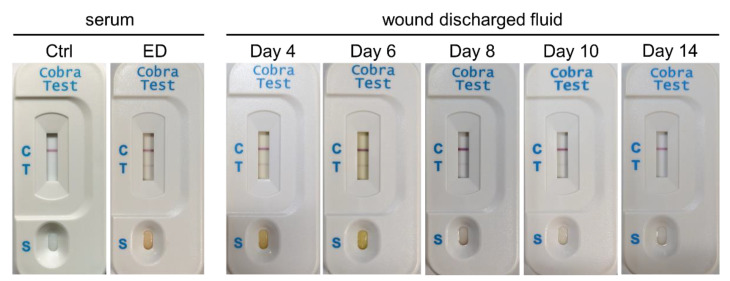
A series of results of the rapid ICT-Cobra test. The serum of a 68-year-old female was collected when she visited the emergency department (ED). Diluted wound discharge fluids were collected on Days 4, 6, 8, 10, and 14. Positive ICT-Cobra tests in wound discharge were noted until day 10, even though advanced debridement was performed on day 6. Ctrl: control serum. The labels on the plastic cassette indicate the control line (C), which is used to check the immunochromatographic function; the test line (T), which is used to detect the presence of venom in the sample; and the sample loading zone (S).

Anti-CTX A3 and anti-sNTX, used for toxins detection and arose from mice have good specificity for CTX A3 and sNTX respectively, have no obvious cross-reactivity with other toxins (Figure S1). From 2016 to 2018, only 4 of 10 cases of *N. atra* envenomation cases developed exacerbated tissue injury. The concentrations of venom and toxins in patient sera, bullae fluids, wound discharge, and subsequent excised necrotic tissues were analyzed using sandwich ELISA and are shown in Table 1. A series of data of the index case (the 68-year-old female, the progression of her local wound was shown in Figure 1) and the results of ICT-Cobra test are plotted in Figure 2 and Figure 3, respectively. In these four cases, case Male/55 was a case of early presentation and antivenom treatment, in contrast to the other three, which we referred to as delayed presentations [15]. All cases suffered from a large venom load during envenomation. Case Male/82 called for medical help even after 24 h and still exhibited a significant concentration of venom (6.2 ng/mL) or toxins (204.7 ng/mL CTX A3 and 958.3 ng/mL sNTX) in the serum (Table 1). Unsurprisingly, he presented with some neurological symptoms and developed local tissue necrosis, as we previously reported [15]. Cases M/82 and M/68 received surgical debridement earlier (52 h and 36 h respectively after being envenomed) with fair outcome.

High concentrations of venom and toxins were observed in the serum, bullae, wound discharge and debrided tissue of Case F/68 (Table 1), and the ICT-Cobra kit could detect venom in wound discharge (Figure 3), in addition to serum, which we reported previously [19,20]. Venom concentration diminished in the serum and decreased after antivenom therapy, but CTX A3 persisted in wound discharge or local tissue until advanced debridement. In contrast to CTX A3, sNTX decreased over time and became significantly exiguous due to wound discharge after various doses of antivenom (Figure 2).

## 3. Discussion

The four patients in this study suffered from *N. atra* envenomation with large venom loading and presented with fewer neurological manifestations in contrast to the notable local dermonecrosis, necessitating multiple surgical interventions and prolonged hospitalization in addition to the high dose (4–10 vials) of antivenom. This observation is not only in line with other reports but also provides quantified data as evidence [21,22,23]. In these cases, the presence of large amounts of venom or toxins were noted and persisted in the wound, including bullae fluid, until the necrotic wound was extensively debrided and excavated, and cytotoxin, such as CTX A3, was the most commonly detected toxin in all wound discharge fluid samples (Table 1). A neurotoxin with a small molecular weight of 6–7 kDa, sNTX was found to be absorbed from the bitten wound and was primarily present in patients’ serum, while only a minor quantity remained in local tissue (Table 1). Our findings in clinical victims are consistent with most animal studies showing that these cytotoxins might represent the major toxin of cobra venom that induces local tissue injury [12,24].

Based on their structure and nonenzymatic functions, cytotoxins and neurotoxins are categorized as three-finger toxins (3FTxs) and are critically involved in envenomation [17,25,26]. All 3FTxs have a similar structure, which consists of four highly conserved disulfide bonds and three distinct beta-sheet loops projecting from the core [27]. These 3FTxs and phospholipase (PLA_2_) have been reported to comprise 70–90% of cobra venom [7,12,28,29]. CTX A3 and sNTX are the representative toxins of cytotoxins and neurotoxins in the venom of *N. atra* [30]. In animal studies, sNTX causes the rapid death of rodents through respiratory paralysis and has been attributed to being the primary toxin causing lethal effects in mice; instead, CTX A3 contributed less lethal effects in animals but proved to be the most abundant and toxic isoform of cytotoxins in *N. atra* venom by an in vitro cell-based penetration assay [12,31]. In domestic studies and further proteomic analysis, cytotoxin was not only the most abundant toxin in the venom of *N. atra*, at 50% or more [7,12,32], but was also found to possess high tissue affinity and resist being metabolized and removed [33,34,35]. Biochemical mapping revealed that the structural loops of CTX A3 present a membrane-binding motif that interacts with the phospholipid bilayer to change the conformation of the cell membrane, forms a transient pore for toxin internalization [36,37,38], and initiates necrotic cell death. With respect to the kinetic data of whole venom, sNTX and CTX A3 in these 4 cases, decontaminating the venom, especially the possible offending toxins and cytotoxins, from local tissues by advanced excision and debridement (Figure 2), in addition to specific antivenom might represent a possible solution for reducing local tissue injuries.

Antivenom is the only antidote available for snake envenomation. The application of specific antivenoms for snakebites has greatly aided in the reduction of snakebite-induced mortality worldwide, including in Taiwan [14,17,39]. Referring to the guidelines of venomous snakebite management in Taiwan [40], neurotoxic antivenom (TNAV), which is raised from horses by immunization with the venoms from *B. multicinctus* and *N. atra*, was suggested to be the only therapeutic option for cobra envenomation. TNAV was found to be highly effective in neutralizing neurotoxins in cobra venom in vitro and in vivo, and clinically preventing lethal respiratory failure in patients and animals [16,21,31], as well as in the case, M/82. Our cases showed that venom levels diminished in the patients’ serum in response to various doses (4–10 vials) of TNAV, as in our previous report [15], and here, kinetic evidence showed that the representative neurotoxin sNTX faded from patients’ serum, local tissue, and wound discharge (Figure 2 and Table 1). However, local tissue destruction accompanied by advanced necrosis remains a critical problem for cobra snakebites, even after 15 vials of specific antivenom [15,18]. Although we found that prompt and correct diagnosis along with early administration of specific antivenom might be valuable for reducing the occurrence of severe tissue injuries [15,20], several regional reports have still indicated that over 60% of cobra envenomation cases needed additional surgical intervention due to the large area of tissue necrosis occurrence [21,23,41], as in these cases. The occurrence of persistently high amounts of CTX A3 in local tissue and wound exudate and their clearance after deep debridement in our cases indicate that early excision and deep debridement in cases of proven cobra snake envenomation with significant necrotic lesions might be valuable in the prevention of advanced wound destruction. A prospective study addressing these issues is ongoing.

Snake venom phospholipase A_2_ is a small protein with a molecular mass of ~13–15 kDa that is one of the major components of venom and has been suggested to be a factor contributing to dermonecrosis in cobras. The action of cobra venom PLA_2_ requires calcium ions (Ca^2+^) as a crucial cofactor to catalyze the hydrolysis of phospholipids at serine-2 positions to produce lysophospholipids and free fatty acids and might directly destroy the cellular membrane or cooperate with cytotoxins and neurotoxins to potentiate inflammatory and vascular permeability and advancing to irreversible muscle cell damage [42,43]. Generally, two PLA_2_ isoforms with different pharmacological activities are commonly found in snake venoms. The acidic PLA_2_ from Elapidae possesses neurotoxicity, and the basic PLA_2_ from Viperidae exhibits either cytotoxic effects and/or neurotoxicity, depending on the dose [14]. Interestedly, phospholipase A_2_ of *N. kaouthia* and *N. naja* was considered to have the ability to increase cytotoxin-induced cytotoxicity in vitro; however, PLA_2_ from *N. nigricollis* did not exhibit clear cytotoxic effects in an in vivo study, and its inhibition did not reduce the area of necrosis [24,44]. PLA_2_ from *N. atra* has also been found to display more pronounced cytotoxic effects in cancer cells than in normal cells [45]. Even though PLA_2_ accounts for approximately 20% of cobra venom, its cytotoxic role on local envenomed tissue might not be as critical, but this needs more evidential investigation.

Bacterial infection of the wound might also contribute to deteriorated effects on local tissue necrosis [46,47]. The incidence of necrotizing soft tissue infection (NSTI) due to cobra snake envenomation was reported to be less than 20% to more than 70% of predominant Gram-negative bacterial infections with *Enterococcus* spp. or *M. morganii*, which was found in the wounds of our cases, are the most frequently encountered [48]. The synergistic effects of bacterial load in the mouth of the snake, introduced through the fangs and cytotoxic injuries of venom components on local tissues worsen and expand the condition of dermonecrosis to be NSTI. Early radical surgical debridement and empirical broad-spectrum antimicrobial treatment are the cornerstones of NSTI management [49]. In severe cobra envenomation with tissue necrosis, as a consequence, in addition to broad spectrum antibiotic administration to combat invading bacteria, early decontamination with excision and debridement to the extent that is deemed necessary to remove infected tissue and tough toxins might minor the wound, reduce the length of hospitalization, preserve more tissue and prevent disability [48,50].

In this study, we used the ICT-Cobra kit, a rapid test for cobra venom detection, to monitor the wound condition and show promise. The ICT-Cobra kit is designed using a paper-based analytical lateral flow immunoassay, is applied for differential diagnosis of *N. atra* snake bites from other Viperidae snake envenomations [19], and can detect venoms from other Asian cobras with variable detection limits [20]. We used the ICT-Cobra kit to detect venom antigens that were absorbed into circulation after cobra (*N. atra*) bites with a low detection limit (5 ng/mL) and exhibited a strong agreement, including 83.3% sensitivity and 100% specificity with the ELISA results [19]. Here, we used the kit to detect venoms in serum, extended it to assay wound exudate, and revealed it as a simple, rapid, and virtuous method for monitoring the progression of regional wounds, even if residual cytotoxin was the most abundant toxin, not the bulk composition of whole venom. Hence, the ICT-Cobra kit might help physicians quickly identify the severity of local injuries due to cobra bites to implement the correct interventions, such as wound decontamination as soon as possible. In addition to a better prognosis because of early antivenom administration, this rapid test has the potential to minimize the sequences of dermonecrosis.

This is a preliminary report on the possible role of cytotoxin in cobra venom on local tissue pathology, and only four cases were included. Although this might be the first literature has demonstrated that snake toxins persistently exist at high concentrations in local tissues for several days even after systemically administering specific antivenom, there are some shortcomings that should be addressed. First, we were unable to exclude the possible auxiliary role of other toxins in the venom in the local cytotoxic action of CTX A3 because the venom composition was very complex. Second, we collected samples from the gauze that was applied to the wound, isolated the body fluids at various intervals and rinsed it with saline, but the levels of toxins in the wound exudates were difficult to accurately predict. Last, there was some divergence in the venom composition of venomous snakes, the injected dose of each bite, and the time before patient’s sought help in the hospital after being envenomed. Additional case studies or large-scale investigations are needed to clarify the clinical toxicology and adequate management of *N. atra* as well as other cobra snakebites.

Taken together, these limited data provide evidence using human wound exudates and indicate several findings as follows: (1) assaying the concentration of whole venom, or specific cytotoxins, in wound exudate might reflect the progression of dermonecrosis; (2) venom decontamination from earlier debridement might restrict the extension of local tissue damage due to cobra envenomation; and (3) point-of-care tests, such as our ICT-Cobra kit, might be useful for quickly monitoring the wound condition and improving therapeutic outcomes.

## 4. Materials and Methods

### 4.1. Materials and Chemicals

Lyophilized *N. atra* venom was purchased from Latoxan (Portes-lès-Valence, France). Toxins, including cytotoxin A3 (CTX A3) and short-chain neurotoxin (sNTX), were isolated from the venom of *N. atra* (Taiwan) using a high-performance liquid chromatography (HPLC) system (Alliance 2695; Waters, Milford, MA, USA) equipped with a dual absorbance ultraviolet light described in a previous study [31]. Mouse serum anti-CTX A3 and anti-sNTX were raised against the corresponding purified toxins [31]. The horse anti-*N. atra* F(ab’)_2_ was obtained from the Centers for Disease Control (CDC), Taiwan. The horse and rabbit polyclonal antibody was affinity-purified using a Taiwan cobra venom-immobilized column prepared as described in our previous study [51]. Goat anti-horse IgG (H+L)-HRP and goat anti-mouse IgG (H+L)-HRP, the secondary antibodies, were purchased from Jackson ImmunoResearch Inc. (West Grove, PA, USA). The flat-bottom microtitration plates and the TMB substrate were obtained from Corning Inc. (Corning, NY, USA) and SeraCare Life Science Inc. (Milford, MA, USA), respectively. All other chemicals used in this study were analytical grade and purchased from Sigma-Aldrich (St. Louis, MO, USA).

### 4.2. Ethics Statement

Human serum, bullae fluid, excised necrotic tissue and wound discharge samples were used after the patient signed the informed consent form. The use of human specimens was performed after the protocol (protocol No. CMUH107-REC1-005) was reviewed and approved by the Research Ethics Committee (REC) I of the China Medical University and Hospital on 12 February 2018.

### 4.3. Sandwich Enzyme-Linked Immunosorbent Assay (Sandwich ELISA)

The sandwich ELISA was modified and applied to measure the concentration of whole venom (*N. atra*) as well as the levels of CTX A3 and sNTX in serum or wound discharge following the protocol that we previously developed and reported [15,20,51]. One day before analysis, the capture antibody (rabbit anti-*N. atra* IgG, 0.4 µg/mL) was coated onto a 96-well polystyrene microplate with a coating buffer (50 mM carbonate/bicarbonate buffer, pH 9.6) at 4 °C overnight. The next day, the plate was washed five times with 150 µL PBST (0.01% Tween-20 in PBS), and 100 µL blocking buffer (PBST containing 1% BSA) was added per well for a 1 h incubation at 37 °C to block the remaining protein-binding sites. After removing the blocking solution, 100 µL of an already known concentration (0–500 ng/mL) of *N. atra*/CTX A3/sNTX solution and every serum or wound discharge sample were added into wells for another incubation. Following the washing steps for cleaning the residues, primary antibodies, including horse anti-*N. atra* F(ab’)_2_ (10 µg/mL), mouse anti-CTX A3 serum (10 µg/mL), or mouse anti-sNTX serum (50 µg/mL), were added to the wells for another chemical reaction (at 37 °C for 1 h). For substrate conjugation, goat anti-horse-HRP and goat anti-mouse-HRP were loaded and incubated for 1 h at 37 °C. It should be noted that the plate needed to be washed before proceeding to the next step. For the final step, the chromogenic reaction was performed by addition of TMB substrate in the dark for 45 min and terminated by the addition of 1 N HCl. The optical density (O.D.) was measured at 450 nm using a Multiskan FC Microplate Photometer (Thermo Fisher Scientific, Waltham, MA, USA). The concentration of whole venom or each toxin in the samples was calculated by substituting the absorbance value into the standard curve and is shown in nanograms of venom/toxin per milliliter sample (Figure S2).

### 4.4. Cases Presentation and Sample Collection

From 2016 to 2018, a total of 10 cases of *N. atra* envenomation were admitted, and only 4 cases that developed exacerbated tissue injury were included in this study. We collected blood samples when they arrived at our emergency department (ED), and the bullae fluid/wound discharge was collected later. Patients were diagnosed, and their wound condition was monitored using ICT-Cobra kit and assayed the venom or toxins level in serum, wound discharge, or debrided tissue. The wound discharge fluid was collected from the dressing gauze that was applied to the wound every day. During periods of wound progression, we collected wound discharge-cemented gauze, soaked it in 1 mL of normal saline for minutes, centrifuged at 2500 rpm at 4 °C and repeated 3 times. The obtained fluid was transferred into a new centrifuge tube, tested by the ICT-Cobra test and stored at −20 °C for subsequent venom/toxin assay. The concentrations of whole venom or toxins, CTX A3 and sNTX, were determined by sandwich ELISA later. These patients were treated with various doses of specific antivenom according to physicians’ experience and their wound condition that was partly guided by the result of the ICT-Cobra test. To ensure the quality of analysis, each sample was separated into several tubes if available, frozen, and thawed in advance of every test.

A 68-year-old female presented to our EDdue to a snakebite on the left foot 9 h beforehand (Figure 1a) and was found to have cobra envenomation according to the ICT-Cobra test (Figure 2). Three vials of antivenom (specific polyvalent antivenom for *N. atra* and *Bungarus (B.) multicinctus*, TNAV, produced by the Taiwan Centers for Disease Control) were immediately administered intravenously. Incision/drainage was performed, and three more vials of TNAV were used due to wound necrosis and abscess formation 12 h later. The abscess culture was subsequently identified to have *Enterococcus (E.) faecalis*. Additional doses of antivenom were prescribed during the days after (as indicated in Figure 1) due to worsening wounds (Figure 1b) and positive results of the ICT-Cobra test (Figure 2). Deep fasciotomy and excision debridement were performed to remove necrotic tissue on day 6. Three weeks after the snakebite, she received anterolateral thigh free flap reconstruction. She was discharged after 45 days of hospitalization with good wound healing.

A 55-year-old male was bitten by a snake over the dorsal aspect of his left 4th finger and arrived at our ED 30 min later, and TNAV was administered one hour later after a positive ICT-Cobra serum test. Dermonecrosis developed a few hours later, and bullae fluid showed a positive bacterial culture containing *Morganella (M.) morganii*. He received regional fasciotomy and debridement on the 8th day, and his debrided tissue was sent for pathology and toxin analysis. Pathology revealed gangrene with necrotizing inflammation and diffuse coagulative tissue necrosis with suppuration involving the skin, subcutis, and small artery. He received six vials of antivenom, and the wound spontaneously healed.

The other 68-year-old male suffered from a cobra snakebite on his right hand and right big toe and was transferred to our ED 12 h later due to worsening wound condition of his right hand. A large area of dermonecrosis was observed even after 6 vials of TNAV in 12 h. Surgical intervention with fasciotomy and debridement was performed approximately 36 h after envenomation. Bacterial growth of *E. faecalis* and *M. morganii* from bullae fluid and debrided tissue was noted. The patient received a split-thickness skin graft surgery 2 weeks after snakebite with a good recovery.

Another 82-year-old male suffered from a snakebite on his left foot without seeking medical advice for approximately 24 h with progressive swelling below the knee joint, dysphagia, and general weakness. *N. atra* snakebite was proven by a positive ICT-Cobra test on his serum. After administration of 5 vials of TNAV, he received surgical debridement and excision of the necrotic lesion 52 h after being envenomed. No bacterial growth was cultured from the excised tissue. The wound healed well after a rotational flap.

## Figures and Tables

**Table 1:** The concentration (ng/mL) of whole venom and toxins detected in serum, excised tissue, or wound discharge fluid.

		Cases			
Venom or Toxin Measurement		^#^ M/55	M/68	M/82	^#^ F/68
whole venom	S	25.1	197.1	6.2	91.6
	T	64.3	185.9	88.2	-*
	W	94	252.3	82.6	-*
CTX A3	S	6.4	1535.7	204.7	1013.7
	T	65.2	1541.4	14.2	-*
	W	90.3	9124	470.2	-*
sNTX	S	13.4	1958.3	958.3	91.1
	T	12.4	10.3	18.2	-*
	W	4.5	238.3	131.6	-*
ICT-Cobra	S	P	P	P	P
	W	P	P	P	P
days in hospital		16	24	15	45

S: serum sample collected when patient visited ED; T: excised necrotic tissue; W: wound discharge fluids, T & W were collected at different times (indicated in the text) in each case; P: positive. * Multiple data points were collected and shown in Figure 2. # Abbreviation: M for male and F for female.

## Data Availability

Not applicable.

## Supplementary Materials

The following are available online at https://www.mdpi.com/article/10.3390/toxins14020122/s1, Figure S1: The specificity of mouse serum anti-CTX A3 (a) and mouse serum anti-sNTX (b); Figure S2: The standard curve used in calculating the concentration of CTX A3 and sNTX.
