# Supplementary material for: Local Cytotoxic Effects in Cobra Envenoming: A Pilot Study
Source: Toxins (Basel). 2022 Feb 7;14(2):122. doi: 10.3390/toxins14020122 (PMC8877591; doi:10.3390/toxins14020122)
Supplement: Supplementary file 1 [file toxins-14-00122-s001.zip › toxins-1559320-supplementary.pdf]

Supplementary Materials: Local Cytotoxic Effects in Cobra Envenoming: A Pilot Study

Jing-Hua Lin, Wang-Chou Sung, Han-Wei Mu, Dong-Zong Hung.

Indirect enzyme-linked immunosorbent assay (indirect-ELISA)

The toxin with serial dilution (0.4 to 0.05 µg/ml) was dissolved in coating buffer (50 mM carbonate/bicarbonate buffer, pH 9.6) and coated onto a 96-well polystyrene microplate (Corning, USA) at 4˚C overnight. The next day, the plate was washed five times by 150 µl PBST (0.01% Tween-20 in PBS) and added 100 µl blocking buffer (PBST contain 1% BSA) per well for 1h incubation at 37˚C to block the remaining protein-binding sites. After that, removed the blocking solution and then added mouse serum anti-CTX A3 (10 µg/ml) or anti-sNTX (50 µg/ml) into the wells for another incubation (at 37˚C for 1h). For substrate conjugation, goat anti-mouse-HRP was loaded and incubated for 1h at 37˚C. It should be noted that the plate needed to be washed before proceeding to the next step. As the final step, the chromogenic reaction was performed with TMB substrate in the dark for 45 minutes and terminated by the addition of 1N HCl. The optical density (O.D.) was measured at 450 nm by using Multiskan™ FC Microplate Photometer (Thermo Fisher Scientific, USA). The ELISA titer that presented to the specificity was shown as the sample/blank absorbance ratio.

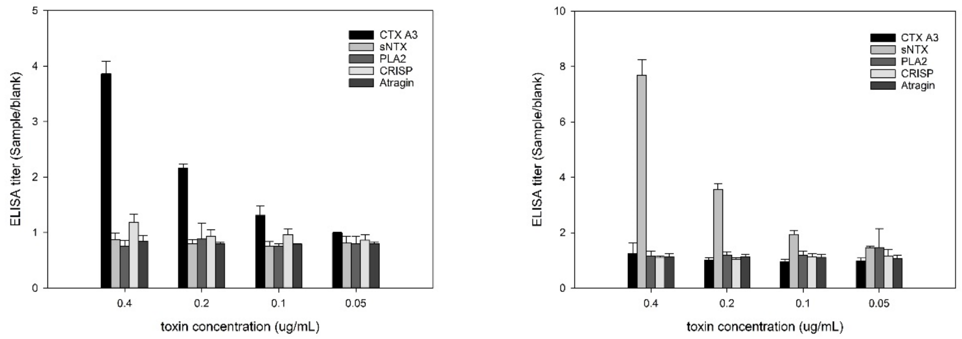

(**a**) (**b**)

Figure S1. The specificity of mouse serum anti-CTX A3 (a) and mouse serum anti-sNTX (b) was confirmed by indirect-ELISA. It was shown that both mouse serum was specific to its own toxin used in the immunization and was nonexistent cross-reaction with other common cobra venom components, including PLA_2, CRISP, and atragin.

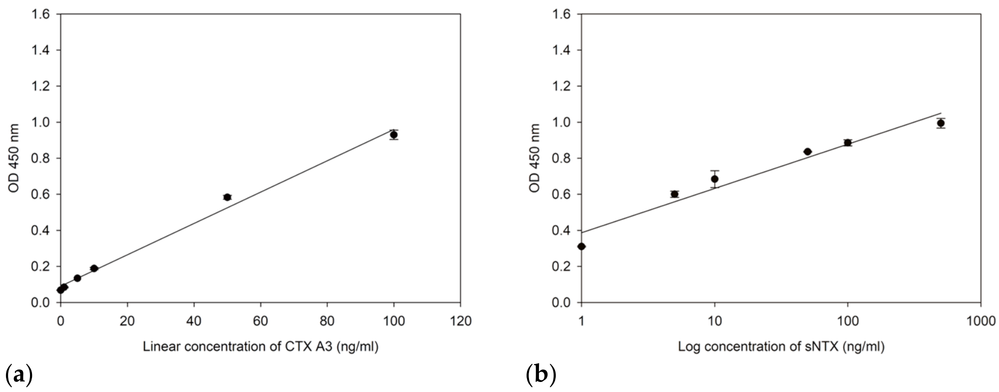

Figure S2. The standard curve used in calculating the concentration of CTX A3 and sNTX. The R^2 value was 0.9918 and 0.952, respectively.
